# Detection of Collapse and Crystallization of Saccharide, Protein, and Mannitol Formulations by Optical Fibers in Lyophilization

**DOI:** 10.3389/fchem.2018.00004

**Published:** 2018-01-26

**Authors:** Jacqueline Horn, Wolfgang Friess

**Affiliations:** Department of Pharmacy, Pharmaceutical Technology and Biopharmaceutics, Ludwig-Maximilians-Universität München, Munich, Germany

**Keywords:** freeze-drying, lyophilization, optical fiber system, glass transition, collapse, crystallization, monitoring

## Abstract

The collapse temperature (Tc) and the glass transition temperature of freeze-concentrated solutions (Tg') as well as the crystallization behavior of excipients are important physicochemical characteristics which guide the cycle development in freeze-drying. The most frequently used methods to determine these values are differential scanning calorimetry (DSC) and freeze-drying microscopy (FDM). The objective of this study was to evaluate the optical fiber system (OFS) unit as alternative tool for the analysis of Tc, Tg' and crystallization events. The OFS unit was also tested as a potential online monitoring tool during freeze-drying. Freeze/thawing and freeze-drying experiments of sucrose, trehalose, stachyose, mannitol, and highly concentrated IgG1 and lysozyme solutions were carried out and monitored by the OFS. Comparative analyses were performed by DSC and FDM. OFS and FDM results correlated well. The crystallization behavior of mannitol could be monitored by the OFS during freeze/thawing as it can be done by DSC. Online monitoring of freeze-drying runs detected collapse of amorphous saccharide matrices. The OFS unit enabled the analysis of both Tc and crystallization processes, which is usually carried out by FDM and DSC. The OFS can hence be used as novel measuring device. Additionally, detection of these events during lyophilization facilitates online-monitoring. Thus the OFS is a new beneficial tool for the development and monitoring of freeze-drying processes.

## Introduction

Freeze-drying is commonly used for the long-term stabilization of biopharmaceuticals which cannot be stabilized adequately in the liquid state. Efficient development of freeze-drying cycles is of utmost importance as the process is time and cost consuming. Short process times without putting the protein stability at risk are desired (Oetjen and Haseley, 2004; Bosca et al., 2013; Pisano et al., 2013). It is essential to analyze the formulation to be freeze-dried regarding critical parameters for the freeze-drying process.

Typical parameters of amorphous matrices, as formed by the most frequently used saccharides for stabilization of proteins, sucrose and trehalose, are the collapse temperature (Tc) and the glass transition temperature (Tg') of the freeze-concentrated solution (Meister and Gieseler, 2009; Pansare and Patel, 2016). They characterize temperatures at which the mobility of the system greatly increases, either at the drying front (Tc) or in the frozen state (Tg'). Tc is typically determined by freeze-drying microscopy (FDM) whereas the standard method for Tg' is differential scanning calorimetry (DSC). FDM mimics the freeze-drying process in miniature by freezing and drying small volumes of formulation under the microscope. Tc can be defined as either the onset of visible collapse or full collapse (Meister and Gieseler, 2009; Bosch, 2014). Tg' as analyzed by DSC reflects the temperature at which the heat capacity of the freeze-concentrated formulation markedly changes (Pansare and Patel, 2016). Both values help to define the upper limit of the product temperature (Tp) during the primary drying step. In lab scale, Tp is typically measured by thermocouples that can only represent the temperature of the surrounding environment although the local temperature of the sublimation front might be more critical for the occurrence of collapse. Tc values are usually 1–3°C higher than Tg' values and drying above Tc may result in macrocollapse of the lyophilizate. Drying above Tg' but below Tc can be utilized e.g., for highly concentrated protein formulations (Colandene et al., 2007). It enables higher product temperatures, faster drying and thus shorter process times without loss of cake structure if protein stability is preserved (Colandene et al., 2007). Nanoparticle suspensions were also shown to increase collapse temperatures (Beirowski et al., 2017). Macrocollapse is not only a question of elegant cake appearance, but it can, but not necessarily has to be correlated to higher residual moisture levels after the process, destabilization of the API, longer reconstitution time or prolonged secondary drying (Chatterjee et al., 2005; Passot et al., 2007; Bosch, 2014). Nevertheless, since it is known that an 1°C increase in Tp can shorten primary drying times by about 13%, the interest is to dry at the highest possible Tp (Pikal, 1990). Crystalline bulking agents like mannitol not necessarily stabilize proteins but form crystalline scaffolds that provide robust and elegant cake structures (Johnson et al., 2002; Hawe and Frieß, 2006; Varshney et al., 2007; Peters et al., 2016). Their controlled and complete crystallization during the freeze-drying process is of interest since partial crystallization might induce subsequent crystallization of the amorphous fraction during storage leading to potential loss of drug stability (Izutsu and Kojima, 2002). Crystallization occurs mainly during thermal treatment before drying starts (Jena et al., 2017). In order to force crystallization, an annealing step is usually conducted at temperatures above Tg' (Liao et al., 2007). The temperature at which crystallization (Tcry) occurs can be determined by DSC measurements (Hawe and Frieß, 2006).

Thus, the correct characterization of the system is of utmost importance. FDM and DSC both provide good approximations but are based on low sample volume which is dried in a thin film by FDM or freeze/thawed in small aluminum crucibles by DSC not necessarily reflecting the several milliliters in a vial during freeze-drying. Furthermore, the high heating rates of 5–20°C/min in DSC analysis do not correspond to the typical 0.5–1°C/min during lyophilization but facilitates the Tg' analysis due to a more distinct baseline shift (Her and Nail, 1994). The heating rates applied in FDM analysis are lower, however, may still not represent the heating rate within the vial (Meister and Gieseler, 2009). The distance from heating source (shelf) to the product combined with the larger sample volume does not lead to a direct transfer of the applied heating rate to the whole product container which slows down the real heating rate in contrast to FDM. The operator itself affects also FDM results as the analysis is performed visually.

Higher Tc values compared to FDM were measured by optical coherence tomography based freeze-drying microscopy (OCT-FDM) indicating that much higher product temperatures can potentially be targeted upon freeze-drying (Greco et al., 2013). In OCT-FDM a single vial freeze-dryer connected to an OCT based camera enables to measure Tc values directly in the product container with the correct filling volume. However, this setup does not represent the conditions of a regular freeze-dryer, e.g., shielding effects of surrounding vials. Crystallization of mannitol and the impact of sucrose could be shown by through-vial impedance spectroscopy (TVIS) (Arshad et al., 2014). This method enables the monitoring of several vials non-invasively and is based on interfacial polarization of glass vials filled with liquid and/or solid. Dielectric analysis (DEA) could also analyze eutectic temperatures of mannitol-water and sodium chloride water system besides the crystallization of mannitol (Evans et al., 1995). There are only few approaches for the determination of the aforementioned characteristics directly in the freeze-dryer (De Beer et al., 2007; Greco et al., 2013; Arshad et al., 2014). Apart from TVIS, De Beer et al. implemented an in-line RAMAN system to detect mannitol crystallization and to differentiate between different mannitol polymorphs (De Beer et al., 2007). The reader is additionally referred to an excellent recent review on the process monitoring devices currently on the market (Nail et al., 2017).

Kasper et al. introduced the optical fiber system (OFS) as potential novel tool to monitor freeze-drying processes (Kasper et al., 2013). The OFS consists of a glass fiber with embedded wavelength dependent reflectors, so called fiber bragg gratings (FBGs). FBGs reflect a certain wavelength of incoming laser light which is detected by an interrogation unit that at the same time acts as light source. The OFS could be established as temperature measuring device by shielding the sensors through embedding in stainless steel tubes. This shielding protects the sensor from forces which occur e.g., during crystallization to which the OFS is sensitive as well besides temperature. However, unshielded OFS sensors may enable the detection of Tg', Tc, or Tcry. Therefore, in this study, temperature and force effects were to be studied by shielded and unshielded OFS sensors. Ultimately, the purpose of this work was to evaluate the possible application of the OFS to analyze collapse, glass transition and crystallization in the product vial in a freeze dryer as well as the potential of the OFS as monitoring device during a lyophilization process.

## Materials and methods

### Materials

Sucrose (Sigma-Aldrich Chemie GmbH, Steinheim, Germany), trehalose (Hayashibara, Okayama, Japan), stachyose (A & Z Food Additives, Hangzhou, China), and D(-)-mannitol (VWR International, Ismaning, Germany) were used in different concentrations (5, 10, or 20% [m/V]) as excipient formulations. The excipients were dissolved in purified water (ELGA LabWater, Celle, Germany). A monoclonal IgG_1_ antibody (mAb) and lysozyme were used as model proteins. 150 mg/mL mAb with 10% (m/V) sucrose in 10 mM sodium phosphate buffer pH 7.0 and 100 mg/mL lysozyme with 10% (m/V) trehalose in purified water served as protein formulations. All formulations were filtered with 0.2 μm polyethersulfone membrane syringe filters (VWR International GmbH, Ismaning, Germany) prior to use.

### The optical fiber system (OFS)

#### OFS unit

**Figure 1:**
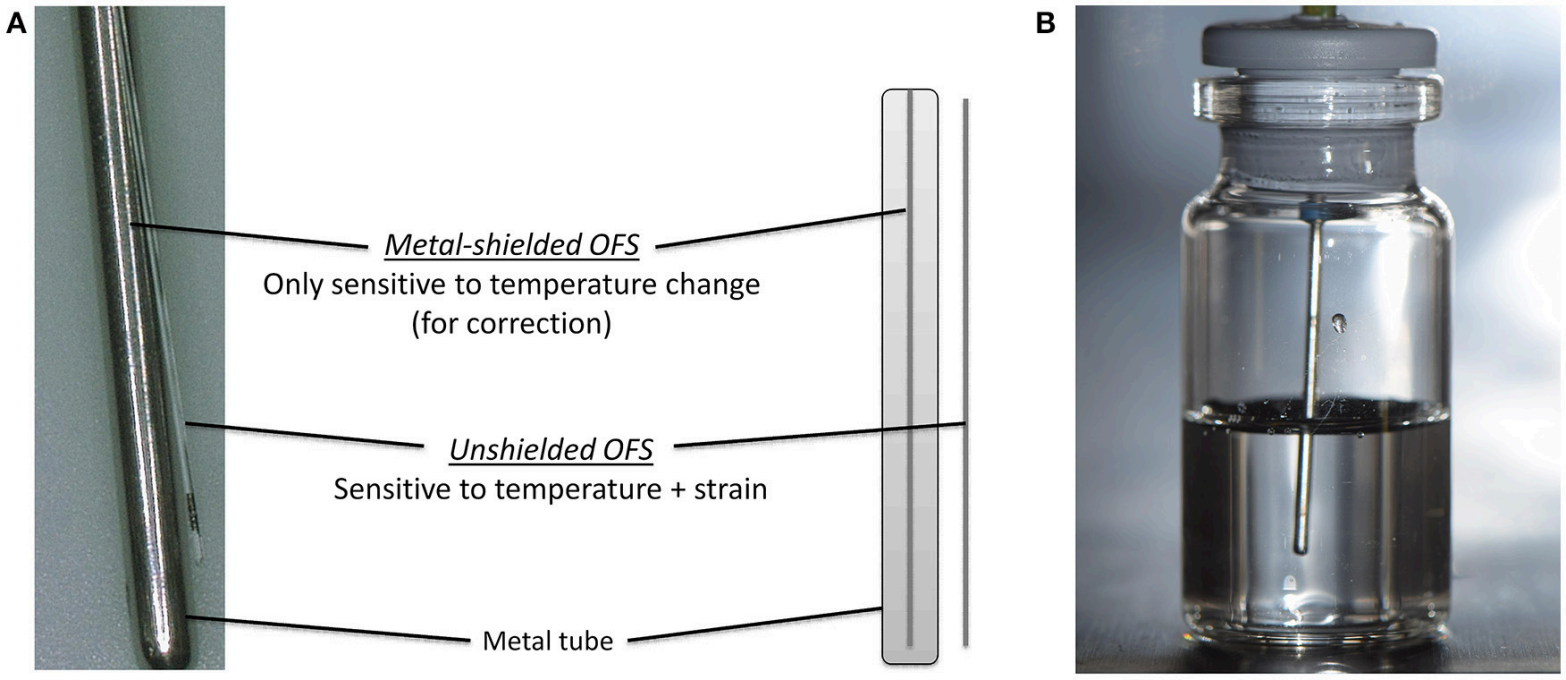
**(A)** Scheme of the OFS unit consisting of shielded and unshielded sensor. **(B)** Placement of OFS unit in a filled glass vial.

The working principle and the design of the used OFS is described elsewhere (Kasper et al., 2013). An OFS unit of two individual OFS sensors was developed (Figure 1A). The temperature was measured by a calibrated, metal-shielded OFS sensor (type: os4210, Micron Optics, Atlanta, GA, USA; T range: −40 to 120°C, short term accuracy ± 0.2°C, response time 0.3 s). Strain induced events were detected by an unshielded OFS sensor (type: fs-FBG, SM1500SC P, polyimide coating removed; INFAP GmbH, München, Germany). OFS data were recorded every 10 s for the complete run time (ENLIGHT 1.0 Sensing Analysis Software by Micron Optics).

6R or 10R glass vials (Fiolax™ clear, MGlas AG, Münnerstadt, Germany) filled with either 4 or 6 mL were stoppered for freeze/thawing experiments or semi-stoppered for lyophilization runs with corresponding lyophilization stoppers (type: Westar®, West Pharmaceutical Services, Eschweiler, Germany). The OFS unit was mounted through the center of the stoppers so that the sensor unit was placed centrally in the solution (Figure 1B). Experiments were performed in triplicates at least if not stated otherwise. Two sensor units were used.

#### OFS–determination of the Tc and Tcry during freeze/thawing

Freeze/thaw cycles between +20 and −50°C at 2.5, 1.0, or 0.5°C/min with 90 min hold times were carried out using a FTS LyoStar™ 3 (SP Scientific, Stone Ridge, NY, USA) freeze-dryer. The wavelength numbers of the unshielded sensor were plotted against the temperature data of the shielded sensor. Wavelength peaks were determined as OFS peak T_OFS_.

#### OFS–use in freeze-drying cycles

Freeze-drying was performed with the FTS LyoStar™. Non-collapse lyophilization cycles were intended to produce lyophilizates with an elegant cake appearance whereas collapse lyophilization cycles should induce a collapse of the lyophilizates (Table 1). The endpoint of primary drying was controlled by comparative pressure measurement (difference ≤ 5 mTorr) between capacitance manometer and Pirani gauge sensor.

**Table 1:** Collapse and non-collapse freeze-drying cycles of sucrose (20%/10%) and stachyose (20%) solutions.

**Freeze-drying cycle**	**Collapse (1)**	**Non-collapse (1)**	**Collapse (2)**	**Non-collapse (2)**
Freezing		−50°C 1°C/min 1.5 h		
Primary Drying	0°C 65 mTorr 0.1°C/min	−25°C 30 mTorr 0.1°C/min	10°C 750 mTorr 1°C/min	−15°C 120 mTorr 0.6°C/min
Secondary Drying	20°C 65 mTorr 0.1°C/min 4 h	20°C 30 mTorr 0.1°C/min 4 h	20°C 750 mTorr 0.05°C/min^−^ 4 h	20°C 120 mTorr 1°C/min 4 h

### Differential scanning calorimetry (DSC)

Tg' and Tcry were analyzed with a Mettler Toledo DSC 821e (Gießen, Germany). 30 μL sample in 40 μL aluminum crucibles were cooled down to −60°C and heated to + 20°C at 10, 2.5, 1.0, or 0.5°C/min. The heating curves were analyzed for Tg'—as the midpoint of the transition—and Tcry peaks.

### Freeze-drying microscopy (FDM)

Collapse temperatures Tc were determined by FDM (Linkam FDCS 196 freeze-drying stage with LinkSys32 software, Linkam Scientific Instruments, Tadworth, United Kingdom) equipped with a Duo 2.5 vacuum pump (Pfeiffer Vakuum GmbH, Asslar, Germany) and an AxioImager A1 microscope used with a 200-fold magnification (Carl Zeiss AG, Oberkochen, Germany). Two microliter of sample was placed on the sample holder together with a spacer and covered by a glass slide. Samples were cooled down to −50°C with 1°C/min. A pressure of 0.1 mbar was applied and the samples were heated to −40 or −30°C with 5°C/min. The sample was held at these conditions to achieve a sufficient thickness of the sublimation front. Tc was detected as onset of collapse in the following drying step at 1°C/min to 0°C. Pictures were taken every 2 s.

### Statistical analysis

Statistical analysis was carried out using SigmaPlot 12.5 (Systat Software GmbH, Erkrath, Germany). One-way ANOVA tests were performed followed by pairwise multiple comparison procedures (Holm-Sidak method).

## Results and discussion

### OFS thermogram

**Figure 2:**
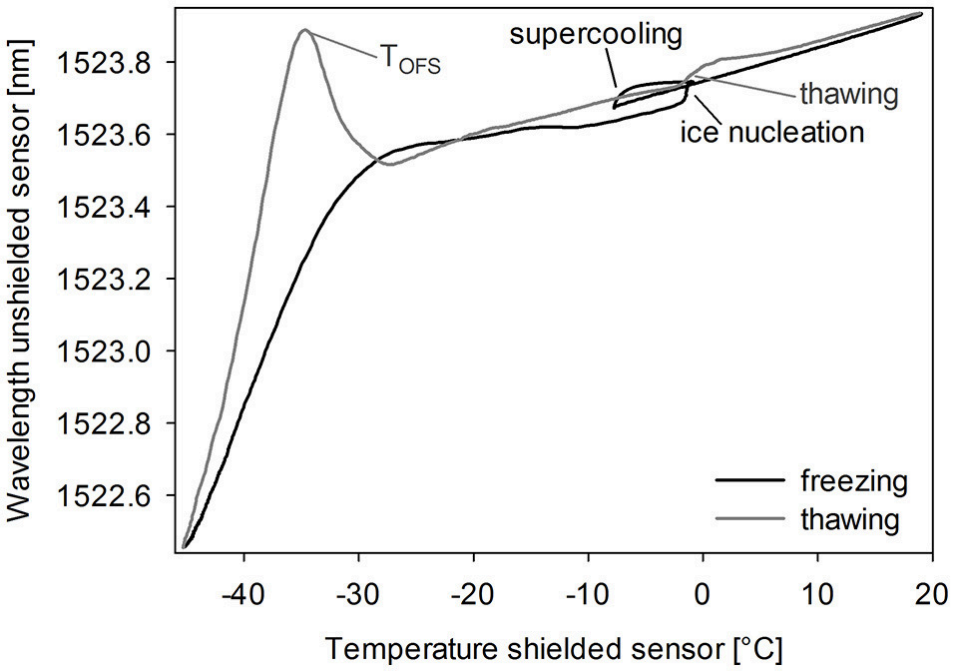
OFS thermogram of sucrose 20% (m/V). Cooling/heating rate 1°C/min. Black line: freezing scan, gray line: thawing scan.

Typically, the unshielded OFS sensor detected several events during freezing and thawing of a sugar solution (Figure 2). Cooling down the solution led to supercooling below the freezing point. As ice nucleation started, many small crystals grew, which did not seem to expose strain on the sensor. The combination of ice crystal growth, solidification of the product and increasing viscosity due to cryoconcentration led to a shift to lower wavelengths with decreasing the Tp to −50°C. During thawing the unshielded OFS sensors showed a pronounced peak close to −30°C, which could be explained by the rapid change in viscosity of the amorphous freeze-concentrate and thus release of mechanical strain (Akyurt et al., 2002; Kasper et al., 2013). This event occurred at a high rate leading to an overshooting peak after which, with rising temperature, the thawing curve approaches the freezing curve. This peak correlated either to Tg' or to Tc. Ice melting led to only a marginal signal and the already reached viscous flow around the sensor might cover up the effect of the event. The OFS shielded by a stainless steel tube did not exhibit this peak but only a temperature profile which corresponded to thermocouple data.

### Detailed investigation of the T_OFS_ peak

**Figure 3:**
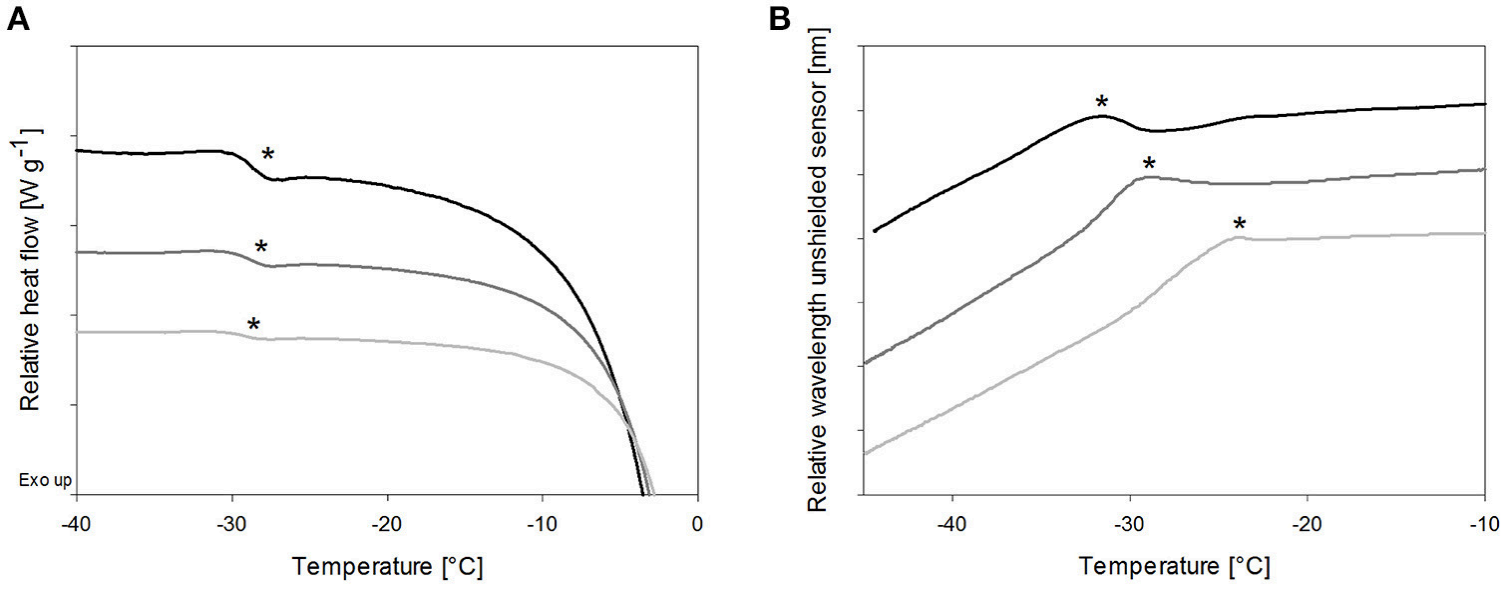
The effect of total solid content of trehalose solutions on **(A)** Tg' in DSC and **(B)** T_OFS_ peaks during heating. Cooling/heating rates were 1°C/min; black line: 20%, dark gray line: 10%, gray line: 5%; ^*^Tg' and T_OFS_ peak resp.

First, it was to be proven whether the T_OFS_ peak at around −30°C resulted from the glass transition and was not an artifact. Therefore, several excipients with different Tg' values were investigated. The two commonly used disaccharides sucrose and trehalose both showed Tg' and T_OFS_ values of around −32 to −28°C (Figure 3). Stachyose, a rarely used tetrasaccharid, showed a Tg' value of −23.8 ± 0.2°C and a T_OFS_ peak at −23.8 ± 0.4°C. Due to the shift of both, the Tg' value and the T_OFS_ peak, to higher temperatures as compared to the discaccharides, the T_OFS_ peak can be attributed to the glass transition of the maximally freeze-concentrated solution or to the collapse. This differentiation was carried out in subsequent experiments (see section Comparison of OFS with DSC and FDM).

#### Impact of heating rate on peak location of T_OFS_

Tg' values obtained in DSC analyses shift to higher values with increasing the heating rate (Her and Nail, 1994; Pansare and Patel, 2016). Typically, in literature heating at 5 to 20°C/min is performed as the Tg can be detected with higher sensitivity and sharpness of the transition as compared to analyzing at lower rates (Coleman and Craig, 1996; Pansare and Patel, 2016). Nevertheless it would be beneficial to measure a more realistic Tg' value at slower heating rates e.g., at 1°C/min. For both sucrose and trehalose solutions, the Tg' shifted to higher values with increasing heating rates in DSC. In contrast, the T_OFS_ peak did not shift between 0.5 and 2.5°C/min (Table 2). The highest DSC heating rate of 10°C/min could not be applied in the OFS setup in the freeze-dryer. In subsequent experiments, 1°C/min was used as default.

**Table 2:** T_OFS_ peaks and Tg' values of sucrose (20%) and trehalose (20%, 10%) solutions at different heating rates.

**Heating rate [°C/min]**	**Sucrose 20%**		**Trehalose 20%**		**Trehalose 10%**	
	**T_OFS_ [°C]**	**Tg' [°C]**	**T_OFS_ [°C]**	**Tg' [°C]**	**T_OFS_ [°C]**	**Tg' [°C]**
10	–	−30.6 ± 0.04	–	−26.4 ± 0.9	–	−27.2 ± 0.3
2.5	−33.8 ± 0.3	−32.3 ± 0.02	−31.3 ± 0.8	−28.5 ± 0.6	−29.1	−29.0 ± 0.2
1	−34.1 ± 0.7	−33.1 ± 0.1	−31.2	−29.4 ± 0.6	−28.8 ± 0.5	−29.9 ± 0.3
0.5	−34.3 ± 0.6	−34.0 ± 0.4	–	−30.3 ± 0.9	−30.5	−30.5 ± 0.4

*Cooling to −50°C was performed with the same rate as heating. Mean values for n = 2, mean ± SD for n ≥ 3*.

#### Impact of solute concentration on peak location of T_OFS_

Cryoconcentration during freezing should end up at the same solute concentration range of the solute in the freeze concentrate, independent of the starting concentration. Nevertheless, higher starting concentrations lead to a more pronounced capacity change at Tg in DSC measurements (Figure 3A, Table 3) which can be explained by the decreasing freeze-concentrate to ice ratio with decreased solute concentration (Her and Nail, 1994). The Tg' value itself was independent of the sugar concentration. Also the sensitivity of the OFS was affected by the disaccharide concentration. The most pronounced peaks resulted at the highest concentration of 20% whereas at the pharmaceutically more relevant solid content of 10 and 5% the peak was flattened and shifted to higher temperature (Figure 3B). The T_OFS_ results for 10 and 20% sugar solutions were similar and close to the Tg' values. The substantially different T_OFS_ for 5% trehalose (−21.1 ± 2.0°C) and 5% sucrose (−24.3 ± 1.4°C) are due to the limited sensitivity of the OFS. In comparison to Kasper et al., the sensitivity of the OFS was improved by to the removal of the polyimide coating (Kasper et al., 2013). But with a lower amorphous phase content the change in force on the sensor is less pronounced and the sensor more in contact with ice. Since the OFS thermograms of 10% sugar solutions showed distinct peaks this pharmaceutically relevant concentration was used for subsequent experiments.

**Table 3:** T_OFS_ peaks and Tg' values of sucrose and trehalose solutions at different concentrations (20, 10, 5%).

**Concentration [% (m/V)]**	**Sucrose**		**Trehalose**	
	**T_OFS_ [°C]**	**Tg' [°C]**	**T_OFS_ [°C]**	**Tg' [°C]**
20	−34.1 ± 0.7	−33.1 ± 0.1	−31.2	−29.4 ± 0.6
10	−29.9 ± 1.0	−33.3 ± 0.2	−28.8 ± 0.5	−29.9 ± 0.3
5	−24.3 ± 1.4	−33.7 ± 0.2	−21.1 ± 2.0	−30.5 ± 0.1

*Cooling/heating rates were 1°C/min. Mean values for n = 2, mean ± SD for n ≥ 3*.

#### Comparison of OFS with DSC and FDM

**Figure 4:**
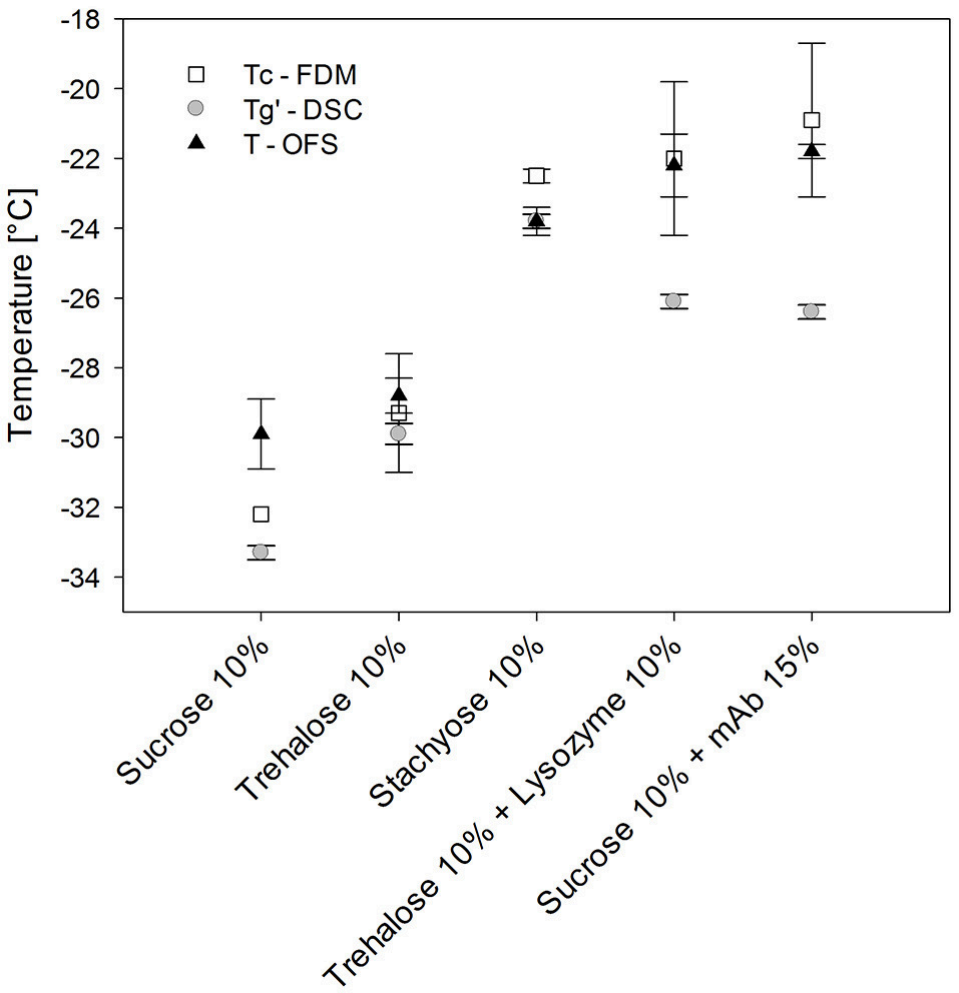
Tg', Tc, and T_OFS_ values of different sugar and protein formulations. Heating/cooling at 1°C/min.

In order to assign the T_OFS_ peak results to either Tg' or Tc, additionally FDM was performed. Sucrose, trehalose and stachyose solutions did not allow to tell since their Tg' and Tc values differed only slightly (Figure 4) (Her and Nail, 1994; Adams and Ramsay, 1996). The Tc and Tg' values of stachyose solutions were within 1.3°C and T_OFS_ was only 1.3°C lower than Tc. Tc and Tg' values of sugar solutions are usually close together (Meister and Gieseler, 2009). Therefore protein solutions were used to tell whether T_OFS_ reflects Tc or Tg'. The presence of protein at higher concentration in disaccharide solutions was shown to lead to a much more pronounced increase in Tc as compared to Tg' (Colandene et al., 2007; Depaz et al., 2016). Consequently, lysozyme 100 mg/mL / 10% trehalose and mAb 150 mg/mL / 10% sucrose were analyzed with OFS, DSC and FDM. Whereas T_OFS_ and Tc did not show a significant difference, Tg' differed statistically significant (*p* < 0.05). Thus, T_OFS_ corresponds to Tc. FDM is based on a subjective evaluation of the onset of collapse. In contrast, the OFS peak is simple to evaluate which helps to avoid differences between various operators, reduces the standard deviation and the OFS unit can easily be placed in the vial filled with formulation.

### OFS application to detect crystallization events

Crystallizing excipients are used in freeze-drying as bulking agents for improved cake appearance since they form robust cake structures. Mannitol is the most preferred excipient for this purpose (Kaialy et al., 2016; Patel et al., 2017). Complete crystallization of the bulking agent after lyophilization is a prerequisite. Partial crystallization during freezing can cause vial breakage during heating due to a secondary crystallization of the non-crystalline fraction and its associated volume expansion (Jiang et al., 2007). Later crystallization upon storage might cause stability problems of the drug (Randolph, 1997). Therefore, it is important to monitor the crystallization process during lyophilization. Typically, crystallinity is analyzed in the lyophilized product by X-ray powder diffraction, DSC measurements, NIR—or Raman spectroscopy (Shah et al., 2006). Mannitol crystallization upon freeze-drying was thoroughly investigated with these methods (Johnson et al., 2002; Liao et al., 2005, 2007; Hawe and Frieß, 2006; De Beer et al., 2007, 2009; Jena et al., 2016, 2017; Peters et al., 2016). Kasper et al. already observed a small peak in OFS thermograms of 5% mannitol solutions during freezing and assigned it as mannitol crystallization (Kasper et al., 2013). However, that OFS was less sensitive due to its coating and a more comprehensive investigation of crystallization events was targeted in the study at hand.

**Figure 5:**
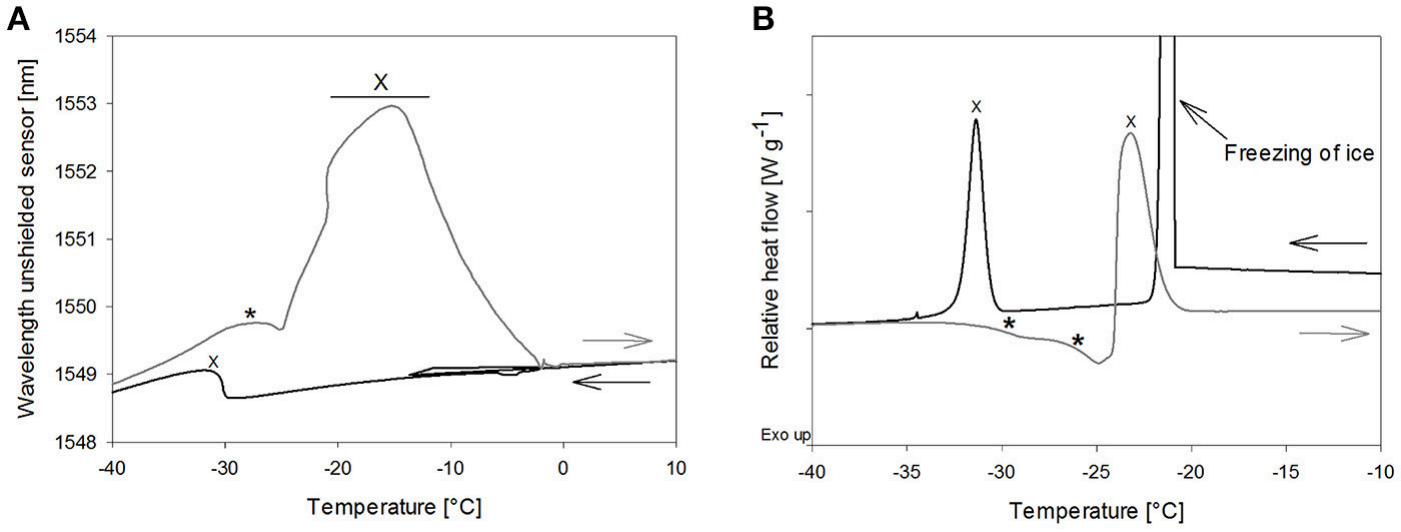
**(A)** OFS and **(B)** DSC freezing and thawing scans of mannitol 10% (m/V). Cooling/heating rate at 1°C/min; black line: freezing curve, gray line: heating curve. ^*^T_OFS_ peaks/Tg'; X: crystallization.

The OFS freezing scan of mannitol solutions showed a broad peak at −32.8 ± 0.8°C, which started to arise at −30°C already and could therefore be attributed mannitol crystallization according to the DSC results (−30.5 ± 0.9°C) (Figure 5). The T_OFS_ peak in the thawing scan at −28.1 ± 0.8°C correlates to Tc of the non-crystalline mannitol fraction. The height of this peak was similar to the T_OFS_ peaks obtained for the amorphous sugar systems (Figure 5A). DSC scans of the mannitol solution rendered two Tg' values in the same temperature range with Tg'^1^: −29.5 ± 0.2°C and Tg'^2^: −24.1 ± 0.9°C (Figure 5B) which corresponded to literature (Cavatur et al., 2002; Hawe and Frieß, 2006; Peters et al., 2016). Thus, the OFS peak can be assigned to an increased viscous flow of the amorphous mannitol fraction.

A pronounced and broad OFS peak starting at −25°C and with a maximum at −16.5 ± 0.9°C was observed during thawing. DSC revealed a second exothermic event at −22.6 ± 0.9°C which was in good agreement with literature values for mannitol crystallization [−19.3°C (Hawe and Frieß, 2006), −22.4°C (Peters et al., 2016), onset at −22°C (Pyne et al., 2002)]. Accordingly, the OFS peak can be attributed to the mannitol crystallization. The height and width of the peak emphasized (1) that the major fraction of mannitol only crystallized in the thawing phase and (2) that mannitol crystallization exerts substantial force onto the OFS sensor. The high forces of mannitol crystallization are known to lead to vial breakage during freeze-drying (Williams and Dean, 1991; Jiang et al., 2007). Thermal mechanical analysis and strain gage techniques demonstrated that volume expansion due to initial crystallization and subsequent crystallization during thawing were the trigger for the vial breakage (Jiang et al., 2007). In our studies with 10% mannitol solutions some experiments ended up in vial breakage too. Vial breakage corresponded to the highest measured OFS peaks. In DSC measurements a 2-fold higher mannitol crystallization peak area was found during thawing as compared to freezing indicating that upon thawing double the amount of mannitol crystallized. Whereas 1% mannitol solutions did not show crystallization in DSC thawing curves and were fully crystalline already after freezing (Peters et al., 2016), the amount of non-crystalline mannitol increases with higher mannitol concentration (10%). Annealing, a typically used hold step above Tg' for complete crystallization of bulking agents, led to disappearance of the mannitol crystallization peak in the OFS thawing curves. This demonstrated again that the peak can be assigned to mannitol crystallization.

Hence the OFS was able to determine phase transitions from solid to viscous flow as well as crystallization of amorphous excipients during freezing and thawing. Thus it can be used as alternative analysis tool to FDM and DSC analysis. The OFS might be more representative for the reality in freeze-drying, since drying rates, freeze-dryer environment, filling volume, and type and size of container correspond to the freeze-drying process.

### OFS application during lyophilization

The ultimate goal was to develop the OFS unit into a process monitoring tool for the freeze-drying process. Kasper already showed that the shielded OFS could be used as a temperature monitoring tool complementary to the usual thermocouples (Kasper et al., 2013). The unshielded sensor may offer additional options. Therefore, two types of uncommon lyophilization cycles were carried out and monitored with the OFS unit. A slow ramp of 0.1°C/min was applied during the start of primary drying to reach a shelf temperature of 0°C in order to induce collapse during this ramp phase [collapse cycle (1)]. For comparison, the same ramp rate but to a lower shelf temperature and at lower chamber pressure was carried out in the non-collapse cycle (1). Faster rates and higher pressure values were necessary to force the occurrence of collapse in stachyose samples due to the higher Tc value [collapse cycle (2)].

**Figure 6:**
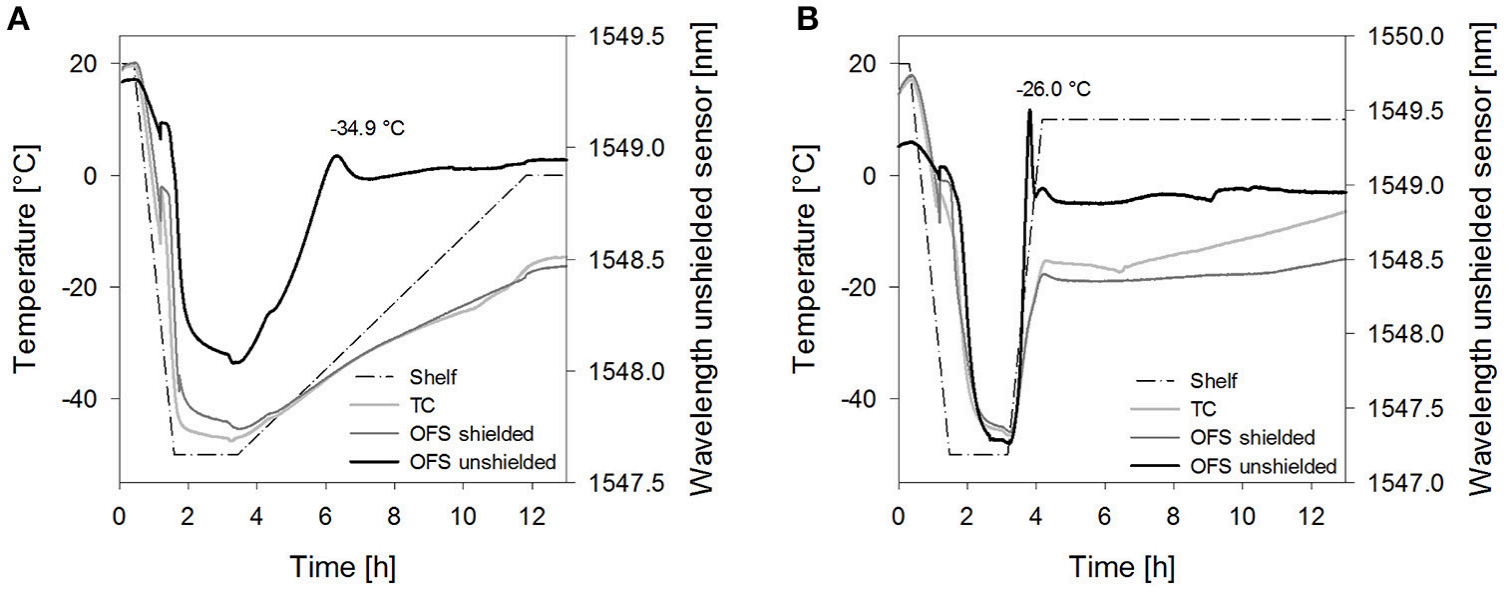
Temperature profiles of **(A)** sucrose 20% samples [collapse cycle (1)] and **(B)** stachyose 20% samples [collapse cycle (2)] obtained with a conventional thermocouple (TC) or an OFS embedded into a stainless steel tube (OFS shielded) in correlation to the unshielded OFS signal. For a better overview, only the first 13 h of the process are shown and pressure values are not included.

The collapse lyophilization cycles demonstrated that collapse can be detected by the unshielded OFS. Both tested saccharides, sucrose 20% and stachyose 20%, showed a peak in the unshielded OFS signal during the primary drying heating ramp (Figure 6). After lyophilization, collapse could be confirmed visually. The location of the peak was associated with the temperature measured by the shielded OFS. Both values were close to the T_OFS_ peaks previously determined in the freezing and thawing experiments. The peak of sucrose was broader and less sharp compared to stachyose which was attributed to the slower heating ramp. Sucrose 20% did not show any OFS peaks or visual signs of collapse when dried utilizing the corresponding non-collapse cycles. For Stachyose 20% a broader transition was noted, which depended on the ramp rate, but no marked peak.

**Figure 7:**
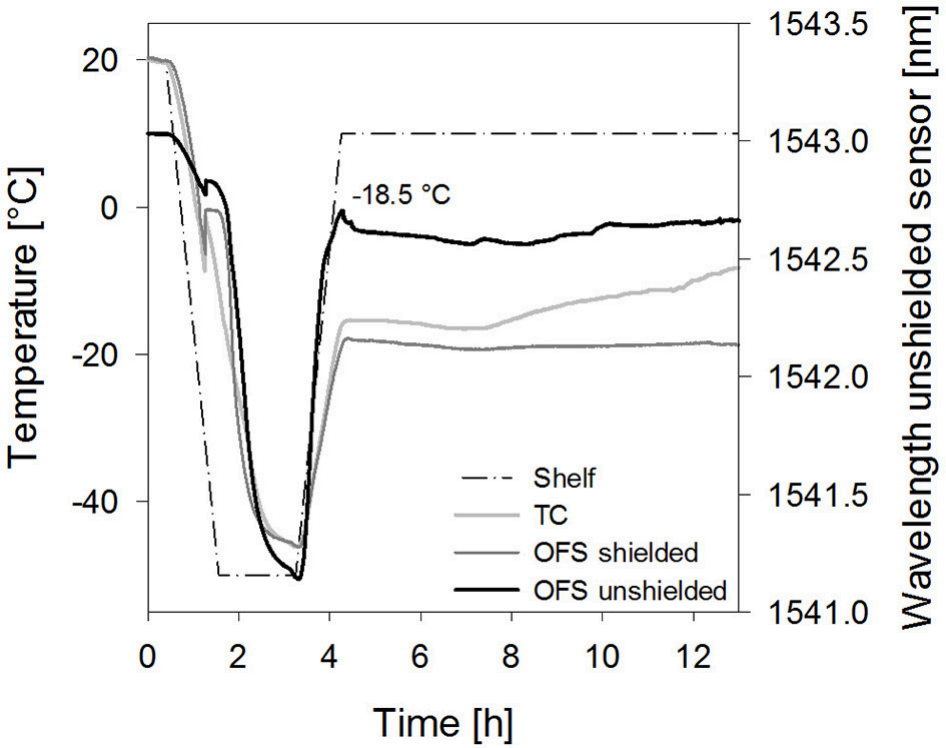
Temperature profiles of sucrose 10% samples [collapse cycle (2)] obtained with a conventional thermocouple (TC) or an OFS embedded into a stainless steel tube (OFS shielded) in correlation to the unshielded OFS signal.

Furthermore, the impact of the solute concentration was investigated. For 10% sucrose samples dried with collapse cycle (2) a smaller peak shifted to higher temperatures resulted with the unshielded OFS (Figure 7). As Tc and T_OFS_ in freezing and thawing cycles were not shifted in DSC at this concentration, the sensitivity of the OFS system may be too low to detect collapse at this concentration at the correct temperature during primary drying. Thus detection of collapse during a freeze-drying run is in general possible, but limited to higher solute concentrations and potential broader transitions may make the evaluation more complex. In order to further develop the OFS as a process monitoring tool, future studies have to focus on an algorithm for detection of peaks and transitions based on the readouts. Formulations with different excipients and concentrations have to be analyzed. This needs to include studies with crystalline bulking agent in combination with an amorphous matrix to learn whether also microcollapse can be detected.

## Conclusion

In this study, the OFS unit could be successfully established as alternative tool for determination of Tc of amorphous systems as well as of Tcry of crystalline phases. The OFS unit combines temperature measurement with the ability to detect force or strain in one device. We could show that the T_OFS_ peak was in good correlation with FDM results. Since the OFS tracks processes that take place in the original sample vial in the freeze-dryer itself the data may be more representative for the product and process development than the data obtained with other instruments like FDM and DSC on manipulated samples. The results furthermore indicate that T_OFS_/Tc values should be set as the upper limit of the product temperature during primary drying rather than Tg' (Colandene et al., 2007; Depaz et al., 2016). The OFS unit is a promising tool that could potentially be developed into a monitoring tool for the entire freeze-drying process. Up to now collapse occurring in the primary drying phase could be detected at high concentrations whereas the desired non-collapse is more difficult to define in terms of the OFS signal. In order to develop the OFS into a process monitoring tool the sensitivity has to be further improved to enable determination of Tc values at lower amorphous excipient concentrations, below <10%. The OFS may also provide further insights into the properties of the freeze-concentrate.

## Author contributions

JH and WF created study design and interpreted the obtained data. JH performed the experiments and the corresponding analysis. The work was drafted by JH and critically reviewed by WF. JH and WF did the final proof of the version to be published and agreed to be accountable for all aspects of the work in ensuring that questions related to the accuracy or integrity of any part of the work are appropriately investigated and resolved.

### Conflict of interest statement

The authors declare that the research was conducted in the absence of any commercial or financial relationships that could be construed as a potential conflict of interest.

## Abbreviations

DSC: Differential scanning calorimetry
FBG: Fiber bragg grating
FDM: Freeze-drying microscopy
OFS: Optical fiber system
PAT: Process analytical technology
Tc: Collapse temperature
Tcry: Crystallization temperature
Tg': Glass transition temperature of the freeze-concentrated solution
T_OFS_: Temperature of OFS peak during heating.
